# *Enterobacter cloacae*, an Endophyte That Establishes a Nutrient-Transfer Symbiosis With Banana Plants and Protects Against the Black Sigatoka Pathogen

**DOI:** 10.3389/fmicb.2019.00804

**Published:** 2019-05-07

**Authors:** Gloria M. Macedo-Raygoza, Benjamín Valdez-Salas, Fernanda M. Prado, Katia R. Prieto, Lydia F. Yamaguchi, Massuo J. Kato, Blondy B. Canto-Canché, Monica Carrillo-Beltrán, Paolo Di Mascio, James F. White, Miguel J. Beltrán-García

**Affiliations:** ^1^Engineering Institute, Universidad Autónoma de Baja California, Mexicali, Mexico; ^2^Department of Chemistry, Universidad Autónoma de Guadalajara, Zapopan, Mexico; ^3^Department of Biochemistry, Institute of Chemistry, University of São Paulo, São Paulo, Brazil; ^4^PPG Ciência Animal, Universidade de Franca, Franca, Brazil; ^5^Department of Fundamental Chemistry, Institute of Chemistry, Universidade de São Paulo, São Paulo, Brazil; ^6^Biotechnology Unit, Centro de Investigación Científica de Yucatán A.C., Mérida, Mexico; ^7^Department of Plant Biology, School of Environmental and Biological Sciences Rutgers, The State University of New Jersey, New Brunswick, NJ, United States

**Keywords:** banana, endophytes, *Enterobacter cloacae*, *Klebsiella pneumoniae*, nutrient-transfer, ^15^N-labeled pheophytin, symbiosis, *Pseudocercospora fijiensis*

## Abstract

Banana (*Musa* spp.) is an important crop worldwide, but black Sigatoka disease caused by the fungus *Pseudocercospora fijiensis* threatens fruit production. In this work, we examined the potential of the endophytes of banana plants *Enterobacter cloacae* and *Klebsiella pneumoniae*, as antagonists of *P. fijiensis* and support plant growth in nutrient limited soils by N-transfer. The two bacterial isolates were identified by MALDI-TOF mass spectrometry and corroborated by 16S rRNA sequence analysis. Both bacteria were positive for beneficial traits such as N-fixation, indole acetic acid production, phosphate solubilization, negative for 1-aminocyclopropane 1-carboxylic acid deaminase and were antagonistic to *P. fijiensis*. To measure the effects on plant growth, the two plant bacteria and an *E. coli* strain (as non-endophyte), were inoculated weekly for 60 days as active cells (AC) and heat-killed cells (HKC) into plant microcosms without nutrients and compared to a water only treatment, and a mineral nutrients solution (MMN) treatment. Bacterial treatments increased growth parameters and prevented accelerated senescence, which was observed for water and mineral nutrients solution (MMN) treatments used as controls. Plants died after the first 20 days of being irrigated with water; irrigation with MMN enabled plants to develop some new leaves, but plants lost weight (−30%) during the same period. Plants treated with bacteria showed good growth, but *E. cloacae* AC treated plants had significantly greater biomass than the *E. cloacae* HKC. After 60 days, plants inoculated with *E. cloacae* AC showed intracellular bacteria within root cells, suggesting that a stable symbiosis was established. To evaluate the transference of organic N from bacteria into the plants, the 3 bacteria were grown with ^15^NH_4_Cl or Na^15^NO_3_ as the nitrogen source. The ^15^N transferred from bacteria to plant tissues was measured by pheophytin isotopomer abundance. The relative abundance of the isotopomers *m/z* 872.57, 873.57, 874.57, 875.57, 876.57 unequivocally demonstrated that plants acquired ^15^N atoms directly from bacterial cells, using them as a source of N, to support plant growth in restricted nutrient soils. *E. cloacae* might be a new alternative to promote growth and health of banana crops.

## Introduction

Bananas (*Musa* spp.) are included in the top five main staple food crops in the world; approximately 100 million tons of bananas are produced annually in approximately 120 countries in tropical and subtropical regions (Ploetz et al., 2015). The sustainable production of bananas is a challenge in many ways due to the use of large amounts of pesticides to control diseases caused by fungi, bacteria, insects and nematodes, as well as the extensive use of fertilizers (Arango-Isaza et al., 2016).

In Mexico and many other countries, the major threat to banana production is black Sigatoka disease caused by *Pseudocercospora fijiensis* (Previously: *Mycosphaerella fijiensis*), which often reduces yield by more than 50%. Infection of banana plants by *P. fijiensis* leads to necrotic streaking on leaves and loss of photosynthetic capacity. Due to the lack of effective host resistance, management of this fungus is based on frequent fungicide applications. Weekly applications in most banana plantations lead to rapid evolution of fungicide–resistant populations (Aguilar-Barragan et al., 2014). Given its importance for food security and likely side effects on human health from pesticide residues on fruits, we need an urgent eco-friendly strategy for protecting banana crops.

Endophytic bacteria are becoming increasingly recognized in crop production because of their potential as agents in plant growth promotion, stress alleviation and biological control as well as their role in making available organic nitrogen sources for plants (Beltran-Garcia et al., 2014b; Santoyo et al., 2016; Maksimov et al., 2018; White et al., 2018). The direct growth promotion by endophytes has been attributed to the production of plant growth regulators, N-fixation, 1-aminocyclopropane 1-carboxylic acid (ACC) deaminase activity and phosphate solubilization. Diazotrophic endophytes are versatile microbes and can provide nutrients to plants even though they lack nodules, a process called ‘associative nitrogen fixation’ (Carvalho et al., 2014).

In this study, we addressed the potential application of two endophytic bacteria that were isolated from leaves and roots of banana plants cv. ‘Cavendish Grand Naine,’ with capacities to support the growth of their host plant in extremely poor soil nutrient conditions and antagonistic activity against the black Sigatoka pathogen. However, our main interest was to assess whether these endophytes support banana plant growth in soils with limited nutrients.

## Materials and Methods

### Biological Material

#### Bacteria

The *Escherichia coli* (ATCC^®^ 25922^TM^) was purchased from ATCC (Manassas, VA, United States) and was used as non-endophyte for plant growth assay and nitrogen transfer experiments.

#### Fungi

Two strains of *P. fijiensis*, *Mf-1* (sensitive to fungicides) and *102* (resistant to fungicides) isolated in Mexico from diseased banana leaves ‘Cavendish Grand Naine’ was used for antagonism assays. Identification was carried out by PCR techniques using the specific primers for β-tubulin (Anzarlou et al., 2007) on single-ascospore strains. Comparison of phenotypes and fungicide resistance of these strains was previously reported by Beltrán-García et al. (2014a).

#### Plants

Micropropagated plants of banana (French clone) were purchased from Nature Source Improve Plants of Mexico (Frontera Hidalgo, Chiapas, Mexico).

### Isolation of Endophytic Bacteria

Ten individuals of banana cv. ‘Cavendish Grand Naine’ without black Sigatoka disease were randomly collected in commercial plantations of Colima and Jalisco states, Mexico. Fresh 5 cm^2^ leaves and 5 g roots were washed with tap water and surface sterilized with 3% commercial Clorox for 10 min and then with 85% ethanol for 3 min, rinsed three times with sterile distilled water until eliminate hypochlorite. “Sterility” test was carried out by using 0.1 mL of the last wash and spread it onto Trypticasein Soy Agar or TSA (Sigma-Aldrich, Mexico) plates. The plant material was macerated with 0.9% NaCl in mortar and pestle under aseptic conditions. One hundred microliters were plated on TSA and formulated Norris medium (Atlas, 2004), and then incubated at 30°C for 10 days to isolate diazotrophic bacteria. The bacterial colonies were selected, sub-cultured and purified. Bacterial morphology was determined by a light microscopy, and Gram staining was tested.

### Bacterial Identification

The identification of the bacterial strains was performed by matrix-assisted laser desorption ionization (MALDI) time-of-flight (TOF) mass spectrometry (MS). To generate spectra for strain identification, the bacterial strains were cultured in TSA during 18 h at 30°C. The samples were analyzed in a mass spectrometer MALDI-TOF (AutoFlex Speed, Bruker; Bremen Daltonics, Germany). Sample preparation methods were performed as recommended by the manufacturer’s protocol. To generate spectra we select a linear positive method from 5 to 20 kDa, with 35% of the laser and acquiring 2000 laser shots for the subsequent analysis. The bacterial identification was performed in MBT COMPASS MALDI BIOTYPER (Bruker; Bremen, Germany). Each target plate was externally calibrated using spectra of the reference strain *Escherichia coli* DH5α (Bruker; Bremen, Germany).

Bacterial identification was corroborated by PCR amplification and sequencing of 16S rRNA. For PCR reactions, the following primers were used: **27f** 5^′^-AGA GTT TGA TCC TGG CTC AG-3^′^ and **1492R-Y** 5^′^-GGY TAC CTT GTT ACG ACT T-3^′^, corresponding to V5 region of the gene which produce an amplicon with ∼1500 bp. The PCR reaction was conducted using the following conditions: preheating (94°C, 5 min), followed by 35 cycles of denaturing (94°C, 30 s), annealing (55°C, 45 s) and extension (72°C, 40 s) and final extension (72°C, 5 min) (Thomas, 2004). PCR products were separated using 1% agarose gel, and sent to the Molecular Biology Unit in the Cellular Physiology Institute, UNAM (Mexico City, Mexico) for sequencing. A phylogenetic tree was constructed using the Neighbor-Joining method (Saitou and Nei, 1987). The optimal tree with the sum of branch length = 1.43783772 is shown. The evolutionary distances were computed using the Jukes-Cantor method and are in the units of the number of base substitutions per site. The proportion of sites where at least 1 unambiguous base is present in at least 1 sequence for each descendent clade is shown next to each internal node in the tree. The analysis involved 11 nucleotide sequences. Codon positions included were 1st + 2nd + 3rd + Non-coding. All ambiguous positions were removed for each sequence pair. There were a total of 1513 positions in the final dataset. Evolutionary analyses were conducted in MEGA7 (Kumar et al., 2016).

### SEM Analysis of the Endophytic Bacteria From Banana and *E. coli*

Bacteria incubated in TS broth (TSB) by 18 h at 30°C, were centrifuged at 4000 × *g*, 4°C for 15 min. The supernatant was discarded, and the pellet washes three times with 15 mL of sterile water and centrifuged as above. Pellet was carefully suspended in 25 mL of sterile water (mixed with pipette tip), and 1 ml was transferred to 2 ml Eppendorf tube and centrifuged at 16000 × *g* for 2 min. The cells were fixed in 2.5% glutaraldehyde for 1 h followed by 4 washes with 0.1 M cacodylate buffer pH 7.2 for 1 h each. Samples were then gently placed in 1% osmium tetroxide in 0.1 M cacodylate buffer for 1 h, washed three times with cacodylate buffer as before, then added with 1% tannic acid for 30 min and washed twice with deionized water. A second fixation with 1% osmium tetroxide for 30 min was performed and washed three times with deionized water. Then, the samples were dehydrated in a series of increasing concentrations of ethanol (10% steps) for 10 min each. After critical point drying, samples were sputter-coated with a thin (10 nm) layer of gold and stored dehydrated until analyzed in a FEI Quanta FEG 250 scanning electron microscope using 5 KV (FEI Company, Hillsboro, OR, United States).

### Characterization of Endophytic Bacteria

#### Antagonistic Activity on *P. fijiensis* Strains

**FIGURE 1:**
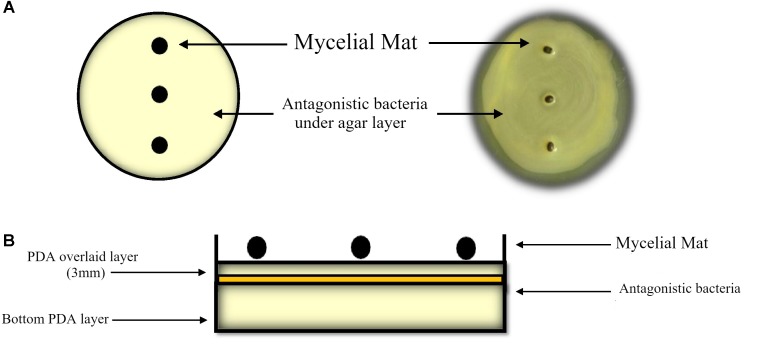
Antagonistic assay by the agar double layer method: **(A)** Top view, **(B)** side view of the first agar layer (bottom) to support bacterial growth and biofilm development after 24 h. A three mm PDA layer (overlaid) is placed onto bacterial grown as top layer for mycelial mat grown.

To determine antagonism of endophytic bacteria to growth of the fungicide sensitive *P. fijiensis* (MF-1) strain and the fungicide resistant strain 102 (both described in Beltrán-García et al., 2014a), the double agar layer or sandwich method was used. Bacteria were grown in TS broth until they reached the mid-log phase at 16 h. Bacterial cultures of *E. cloacae* and *K. pneumoniae* were adjusted to Abs_600 nm_ = 1 equivalent to 1.84 × 10^6^ and 1.1X10^6^ CFU/mL respectively. Aliquots of 200 microliters of bacterial cultures were spread with a cotton swab all over the Petri dish with Potato Dextrose Agar (PDA). Immediately a 3 mm PDA layer was overlaid onto inoculated agar, so that bacteria do not come into direct contact with the mycelial mat, then incubated at 30°C for 24 h. Mycelial pellets 3 mm in diameter were inoculated on the agar (as shown in Figure 1). Fungal colony diameters were measured for 7 days, and the percent inhibition for each treatment was calculated considering fungal growth on bacterium-free PDA. The mycelial pellets without apparent growth were immersed in streptomycin sulfate solution (5 mg/ml) for 20 min, washed with sterile water and then transferred to a Petri dish with PDA. After 5 days of incubation, mycelium that did not grow was considered to be dead. These analyses were carried out with two replicates per treatment and the experiment was carried out three times.

#### Screening for Nitrogen Fixation

The assay to determine whether endophyte strains fix nitrogen was according to Dobereiner (1995), by using nitrogen-free-malate (NFB-malate) medium was used and bacteria were incubated for 3 days at 37°C. A positive result was evident when growth of colony and change of coloration from green to blue occurred on NFB-malate medium. To confirm the ability of the strains to fix atmospheric nitrogen, seven consecutive streaks were performed on the semisolid NFB-malate medium.

#### Screening for Indole-3-Acetic Acid Production

The quantification of the indole-3-acetic acid was performed according Sawar and Kremer (1995), with modification. Bacteria were incubated at 30°C for 24 h on 50%-TSA. Bacteria biomass was suspended in sterile distilled water, adjusted to DO_500 nm_ = 0.5 and 1 mL was added to 14 mL of 50%-TSB with 5 mM of L-Tryptophan. The bacterial suspension was incubated at 30°C for 72 h in darkness without agitation and then centrifuged at 1792 × g for 20 min at 4°C, and 2 mL of the supernatant were transferred to a Falcon tube and 2 mL of Salkowsky reagent was added (Gorden and Weber, 1951). The mixture was incubated 45 min at room temperature and then measured at 530 nm in a UV-VIS spectrometer (Perkin-Elmer, Lambda 25). The quantity of IAA was determined by comparison with an IAA standard curve and expressed in μg mL^−1^.

#### Phosphate Solubilization

The ability of the bacterial strains to solubilize inorganic phosphate was assayed using a modified NBRIP medium supplemented with hydroxyapatite, as phosphate source (Nguyen et al., 1992; Nautiyal, 1999). The bacteria were inoculated as spots in the center of the Petri dishes and then incubated by 15 days at 30°C. Phosphate solubilization produces a clear halo.

#### Siderophores

Production of siderophores was determined according to Schwyn and Neilands (1997). Both solutions were mixed and poured into Petri dishes. Bacterial strains were inoculated by puncture and incubated at 30°C for 6 days. Medium change color from blue to yellow-orange is indicative of siderophore production.

#### ACC Deaminase Production

The ACC deaminase production was evaluated on minimal media containing ACC as nitrogen source, as described by Penrose and Glick (2003). After two washes with 0.9% saline solution, bacteria were inoculated onto DF salts minimal medium (Penrose and Glick, 2003). The bacterial cultures were incubated for 4 days at 30°C at darkness. Growth on this medium was considered a positive result.

### Pot Experiments to Evaluate Plant Growth Stimulation in Nutrient-Limited Beach Sand

Micropropagated plantlets of *Musa acuminata* were removed from the agar, washed with sterile water, placed in 150 g of sterile sand and then incubated at 50% humidity, 30 ± 5°C and 1000 lux light (14 h light and 10 h dark) by 15 days for acclimatization. Each treatment consisted of 4 pots with two plants (8 total plants per treatment). The acclimatized banana plantlets were subjected weekly for 60 days to the following treatments: (1) water control, where plantlets were watered with 5 ml of sterile distilled water; (2) mineral solution control, where plantlets were watered with 5 ml mineral solution or MMN [NH_4_Cl 0.5 g/L, CaCl_2_ 0.05 g/L, NaCl 0.025 g/L, KH_2_PO_4_ 0.05 g/L, MgSO_4_^∗^7H_2_O 0.15 g/L, FeCl_3_^∗^6H_2_O 1 mg/L, glucose 5 g/L and 10 ml of trace elements KCl 3.728 g/L, H_3_BO_3_ 1.546 g/L, MnSO_4_^∗^H_2_O 0.05 g/L, CuSO4 0.0125 g/L, and (NH_4_)_6_Mo_7_O_24_^∗^4H_2_O 0.05 g/L] (Behie et al., 2012); (3) endophytes *E. cloacae* and *K. pneumoniae* treatments, where both endophytes were cultured in M9 medium (Na_2_HPO_4_ 6 g, KH_2_PO_4_ 3 g, NaCl 5 g, nitrogen source 3 g, glucose 30 g, and MgSO_4_^.^7H_2_O 0.2 g per liter, with a pH adjusted at 7.4) (Rédei, 2008). We used NH_4_Cl for *E. cloacae* and NaNO_3_ for *K. pneumoniae* as nitrogen sources; and (4) non-endophyte *E. coli* treatment. The *E. coli* strain was grown in M9 broth using NH_4_Cl (Sigma) as nitrogen source. Bacterial cells were incubated by 18 h with rotary shaking at 150 rpm and then cells in the culture medium were harvested by centrifugation at 5000 rpm for 15 min, and washed three times in a 0.05% glucose solution and centrifuged again. For all bacterial treatments, each banana plant were inoculated weekly with 5 mL of bacterial cells suspended with sterile glucose solution (0.05%) at OD_600_ = 1, equivalent to 1.84 × 10^6^, 1.1 × 10^6^, and 2.47 × 10^6^ CFU/mL for *E. cloacae*, *K. pneumoniae*, and *E. coli* respectively. After 60 days, plants from all treatments were collected to measure growth parameters such as plant length, numbers of leaves, fresh plant weight, and for pheophytin extraction. The plant experiments were done two times.

### Measurement of ^15^N Transference From Selected Bacteria to Pheophytin in Banana Plantlets

To evaluate transference of organic N from bacteria to plants under limited nutrient conditions, *E. cloacae* and *E. coli* were ^15^N-labeled by growing them on minimal medium M9 with ^15^NH_4_Cl (Sigma), *K. pneumoniae* was grown with ^15^NaNO_3_ (Sigma). Bacterial cells were grown for 18 h at 200 rpm, recovered by centrifugation (6000 × *g*, 20 min, 4°C) and washed three times in sterilized glucose solution (0.05%). Plants were inoculated with bacteria as was previously described for plant growth experiments. Plants were grown for 2 months in glass bottles with 150 g of sterile sand beach in a growth chamber at 32°C day/24^°^C night temperature, 14 h light: 10 dark hours photoperiod.

Pheophytin, a magnesium-free derivative of chlorophyll, which has advantages over chlorophyll for isotopic analysis because of its stability, simpler mass spectra and better ionization characteristics (Kahn et al., 2002), was measured as biomarker for transference of nitrogen from endophyte bacteria to plant tissue. An analysis of mass spectrometry in high-resolution was carried out to evaluate the relative abundance of isotopomers of pheophytin. Fresh leaves (0.1 g) from plants under all treatments were frozen in liquid N_2_ and ground to a fine powder using mortar and pestle. For pheophytin extraction, equal volumes of 85% acetone in 1 mL water, and ethyl ether, were added and centrifuged at 11,200 × *g* for 5 min. The ether phase was collected, and the procedure was repeated two times. Chlorophyll was converted to pheophytin by adding 10 μL of 6M HCl to the ether extract. The excess of HCl was removed by adding 500 μL of water, mixing and centrifuging at 11,200 × *g* for 10 min, three times. The ether phase was collected, dried, solubilized with methanol to final concentration of 1 mg/mL. Pheophytin was analyzed using a MicroTOFQ-II mass spectrometer (Bruker, Bremen, Germany) coupled to a Shimadzu HPLC system (Tokyo, Japan) with two pumps LC-20AD, automatic injector SIL-20A, column oven CTO-20A, UV detector SPD-20A, and controller CBM-10A. A column Phenomenex^®^ Luna 5 μm (PFP2 150 × 2 mm, 100A particle size) was used and chromatography was performed with a flux of 200 μL/min using acetonitrile: H_2_O (+0.1% formic acid) as mobile phase in a gradient of 0–5 min 60% of acetonitrile, from 5 to 30 min 60–100% of acetonitrile. The column oven was kept at 40°C, UV detector was recorded at 400 and 600 nm. The mass spectrophotometer was operating in electrospray positive mode, with a nebulization and drying gas at 4 Bar and 8 L/min, respectively. Capillar voltage was set to 4500 V and drying temperature in 200°C. Collision cell and quadrupole energy were set to 20 and 10 eV, respectively. The molecular formula of pheophytin *a* is C_55_H_74_N_4_O_5_ and the base peak was detected at [M+H]^+^ = 872.5731.

### Statistical Analysis

The data were analyzed using the statistical program STATGRAPHICS version centurion XVI (Statpoint Technologies Inc., The Plains, VA, United States). The data for plant growth parameters were analyzed by one-way analysis of variance (ANOVA) with multiple range test for comparison of means of plant fresh weight gained, plant height, new leaves and root length. Differences between treatments were considered significant at a probability level of *p* < 0.05. Antifungal activity data were statistically analyzed by Student’s *t*-test using Microsoft Excel 2013, including for the calculation of the means ± standard errors.

## Results

### Cell Morphology Features and Identification of Endophytic Isolates

#### Endophytic Bacteria Isolation and Characterization

In a previous study, 150 bacterial strains were obtained as endophytes from different tissues of commercial banana Cavendish Grand Naine grown in two different banana-producing regions at central Pacific in Mexico. The bacterial strains were selected for their capacity to grow fast in mineral medium supplemented with colloidal chitin as carbon source; to grow on Norris medium as a way to probe of their N_2_ fixing capacity and grow satisfactorily in culture media based on extracts from different banana plant tissues (unpublished results). Two of the isolates, identified as C2 and RU1E, were selected for this study. Both bacteria are gram-negative, small, rod-shaped, and form colonies that are circular flat (C2), or convex (RU1E) smooth with entire margins, and without pigment production on TSA medium.

**FIGURE 2:**
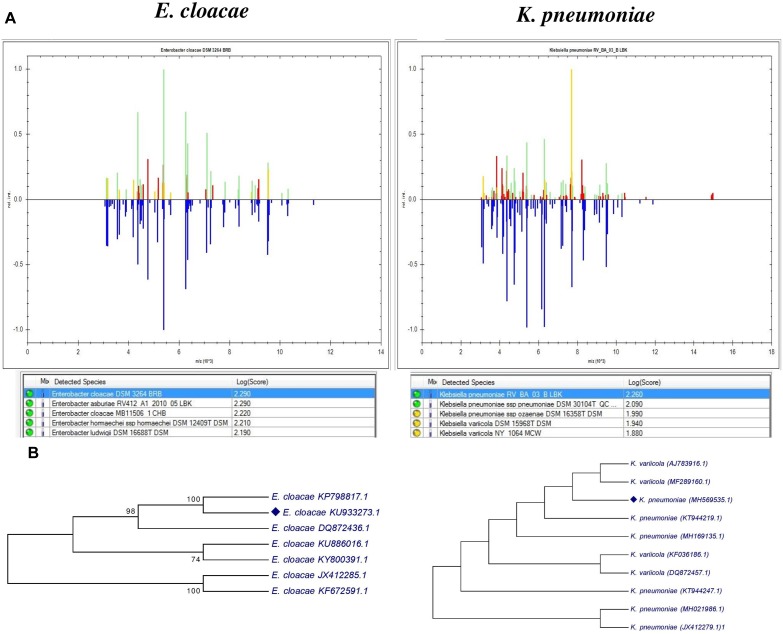
Identification of bacterial endophytes, **(A)** Comparison of mass spectrum protein profiles of bacterial isolates with database of MALDI Biotyper Compass Explorer software version 4.1.60. Blue means the spectrum stored in the database used for pattern matching. Green indicates matched peaks, red mismatched peaks, and yellow intermediate peaks **(B)** phylogenetic analysis of 16srRNA sequences of the two endophytic strains and their comparison with another reported banana endophytic strains. The analysis was conducted with MEGA7 using neighbor-joining method.

For identification, we used MALDI-TOF mass spectrometry and the 16S rRNA sequencing PCR product. Recently MALDI-TOF/MS has been considered as an excellent tool in different research laboratories for detection and discrimination of various types of microorganisms (Pavlovic et al., 2012; Elbehiry et al., 2017). The Figure 2A shows the mass spectrum protein profiles of bacteria and the results were validated with reference strains stored in the database of MALDI Biotyper Compass Satellite Software^TM^ (Bruker) and shows that C2 is *Enterobacter cloacae* (score 2.29) and RU1E is *Klebsiella pneumoniae* (score 2.26). The result of the BLAST analysis of the 1200 and 800 bp long 16S rRNA sequences confirms that C2 isolate is closely related to *Enterobacter cloacae* and the RU1E is a *Klebsiella pneumoniae* respectively. The phylogenetic tree clearly showed that *E. cloacae* (C2) shared 100% similarity with *E. cloacae* KP7988817.1 (Figure 2A), isolated from shoot tips of banana Cavendish Grand Naine (Sekhar and Thomas, 2015). Moreover, C2 strain clustered with others *E. cloacae* isolated as endophyte from banana plants (Figure 2B). The strain RU1E clustered with *K. pneumoniae* isolated from banana, rice, sugar cane, and maize (Figure 2B). The 16srRNA sequences were deposited in GenBank with the follow accession numbers *Klebsiella pneumoniae* (GenBank: MH569535.1) and *Enterobacter cloacae* (GenBank: KU933273).

**FIGURE 3:**
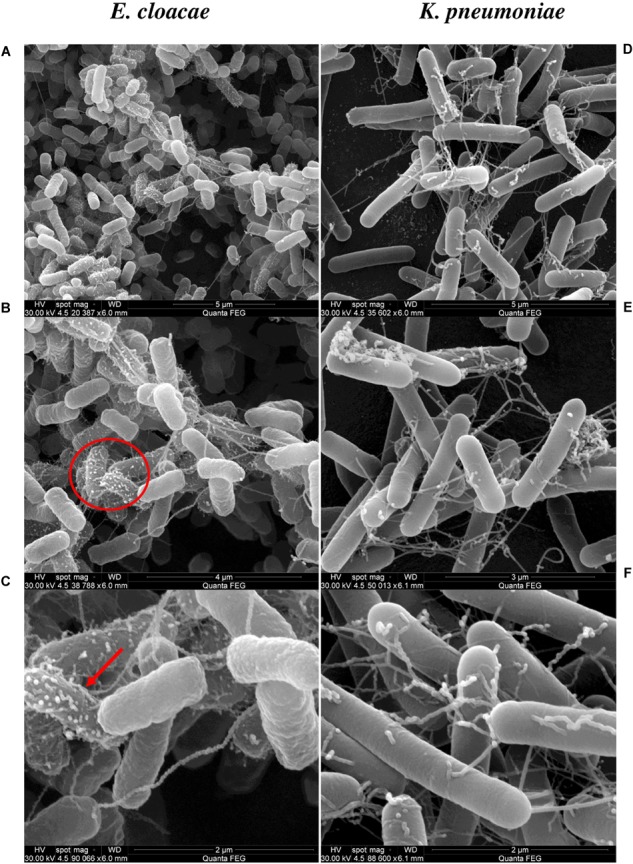
SEM analysis of *E. cloacae* (Ec) and *K. pneumoniae* (Kp) incubated by 18 h. The left and right panels show SEM of Ec and Kp cells, respectively. **(A,B)** Surface of *E. cloacae* cells with lateral flagella, pili. Red circle in **(B)** indicates the area of the next magnification. **(C)** The red arrow indicates formation of outer membrane vesicles (OMV) on the surface of Ec cells, which is not present in Kp all over the bacterial cell at 2 μm. In **(D–F)** *K. pneumoniae* have a larger cell of 0.3–1.0 μm wide X 0.6–6.0 μm lengths, lateral flagella and pili. The bar scales are shown at the lower right of each microphotograph.

The scanning electronic microscopy for *E. cloacae* showed a straight rod-like morphology with cells 0.6–1.0 μm wide X 1.2–3.0 μm long. Cells are bound in clusters (Figure 3A), had a few laterally inserted flagella, a pili and mucoid material that bind cells, possibly some kind of exopolysaccharide (Figure 3B), also with numerous blebs on the surface, suggesting the occurrence of outer membrane vesicles (Figure 3B,C). *K. pneumoniae* shows clusters of cells with rod-like morphology, measuring 0.3–1.0 μm wide and 0.6–6.0 μm in length, possessing lateral flagella and multiple pili; without vesicles (Figure 3D–F).

### Antagonistic Activity of *E. cloacae* and *K. pneumoniae* Against *P. fijiensis*

**FIGURE 4:**
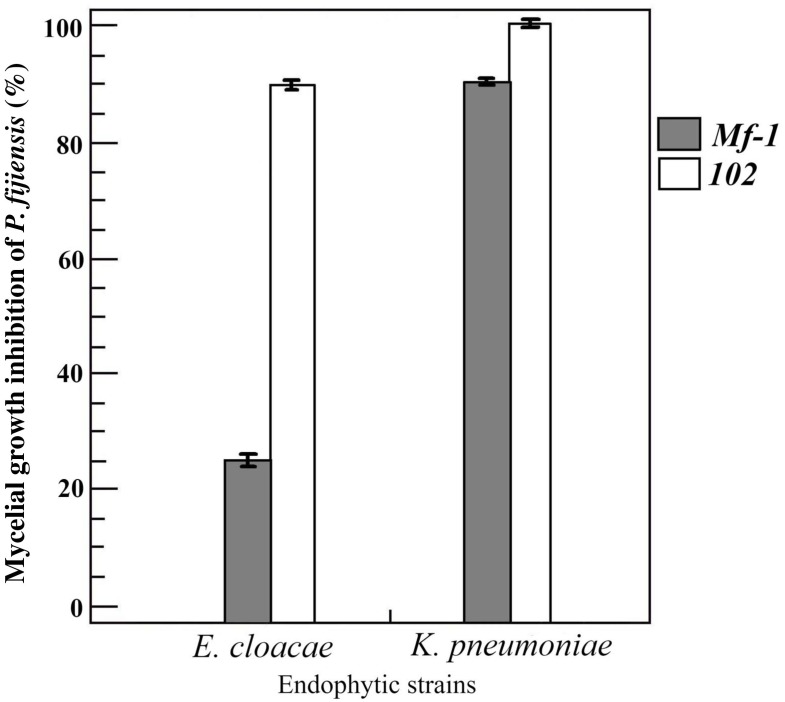
Inhibition of mycelial growth of *Pseudocercospora fijiensis*. The strains Mf-1 (gray bar) and 102 (white bar) were inhibited by banana endophytes *E. cloacae* and *K. pneumoniae*. The *K. pneumoniae* was more antagonistic than *E. cloacae*, especially with the melanized *Mf-1* strain. Bars represent the mean and standard deviation of two experiments with three replications.

The two endophytic bacteria were assayed *in vitro* to measure their antagonistic activity against *P. fijiensis* strains with contrasting sensitivity to fungicides. Both bacteria were antagonistic, but *K. pneumoniae* showed greater inhibition of *P. fijiensis* mycelial growth; the percentage of inhibition exerted against the resistant strain (102) was 100%, while the sensitive strain (*Mf-1*) was 90%. On the other hand, *E. cloacae* showed a 90% inhibition of 102 strain and 25% inhibition of *Mf-1* (Figure 4).

### Plant Growth Promoting Properties (IAA, Phosphate Solubilization, N_2_-Fixation, Siderophores and ACC Deaminase)

Both bacteria produce IAA (Table 1), but *K. pneumoniae* was a poor producer (0.328 μg/mL) in comparison to *E. cloacae* which remarkably produced more phytohormone (74.2 μg/mL). The two bacterial strains were also able to solubilize inorganic phosphate into soluble forms, as they showed a clear zone around the colony. *E. cloacae* was a slightly better phosphate-solubilizer than *K. pneumoniae*; the indexes are shown in Table 1.

*Enterobacter cloacae* and *Klebsiella pneumoniae* were able to grow on the NFb medium (a nitrogen-free medium), developing a biofilm close to the surface; no difference in the degree of turbidity of the medium was observed. On CAS medium *E. cloacae* produced an orange halo after 144 h of incubation, which evidenced the production of siderophores (Pérez-Miranda et al., 2007). The halo color in this test ranges from yellow to orange, indicative of the type of siderophore produced (orange is indicative for hydroxamates) (Bertrand et al., 2009). *K. pneumoniae* produced no siderophores at all (Table 1). Finally, both strains were unable to grow on minimal salt medium amended with ACC, which means that they are not producers of the enzyme ACC deaminase. Unexpectedly, *E. coli* produced IAA (5.47 μg/mL), and was positive for atmospheric nitrogen fixation and ACCd activity; phosphate solubilization and siderophore production were not detected in this *E. coli* strain.

**Table 1:** *In vitro* activities related to plant growth promotion by *E. cloacae* and *K. pneumoniae* strains isolated from banana plants.

Strain	IAA production^∗^ (μg/mL)	Phosphate solubilization Index^∗∗^	N_2_-fixing capacity	Siderophore production	ACCd
*E. cloacae*	74.2 ± 0.04	148.59 ± 5.38	+	+	−
*K. pneumoniae*	0.328 ± 0.020	131.11 ± 7.84	+	−	−
*E. coli ATCC 25922*	5.478 ± 0.5319	ND	+	ND	+


^∗^Average values determined in four replicates samples from cultures on 50%TSB liquid medium supplemented with 5 mM tryptophan. ^∗∗^Averages of solubilization index determined in three replicated cultures on PKV agar plates supplemented with hydroxyapatite. Solubilization index (SI), diameter of the colony plus halo zone/diameter of the colony. ACCd, 1-Amino Cyclopropane-1-Carboxylate deaminase. ±, standard error (SE); +, positive; −, negative; ND, non-detected.

### Growth Support by Endophytic Bacteria to Banana Plantlets Under N-Limited Conditions

**FIGURE 5:**
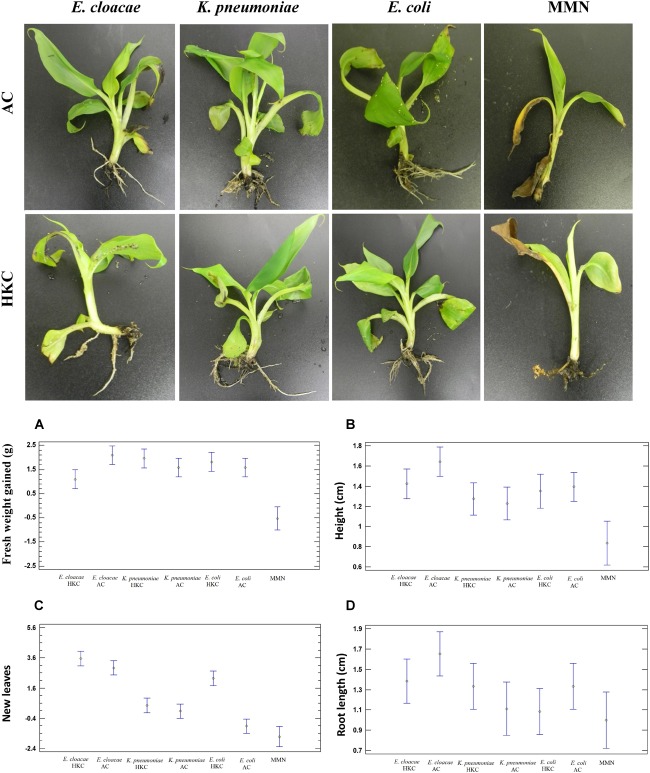
Growth support of banana Cavendish treated with endophytic bacteria and non-endophyte *E. coli* in low nutrient soil microcosm within 60 days. Data are represented are means of two replicates: each replica consisted of five plants. Upper: Representation of banana plant health and root system inoculated with active cells (AC) and heat-killed cells (HKC) and Mineral Nutrient Solution (MMN). Down: Plots illustrating statistical analysis of plant growth parameters such as Fresh Weight Gain **(A)**, Plant Height **(B)**, New leaves **(C)**, Root Length **(D)**. Comparison between treatments was carried out by one-way analysis of variance (ANOVA). Differences were considered significant at a *P* < 0.05 at 95% level of significance.

Plant microcosms were watered with native endophytic bacteria, and ATCC strain of *E. coli* as a non-endophytic bacterium (control) every week. In addition, MMN mineral solution and water were used as positive and negative controls. The beach sand was free of organic material. According to Figure 5, a noteworthy increase in all tested parameters was observed in the bacteria-inoculated plants compared with MMN plant treatments. Also, plants were irrigated with distilled H_2_O (data not shown) and the majority suffered tissue deterioration, chlorosis and died after 2 or 3 weeks. The plants treated with MMN solution showed a loss of biomass (−0.525 g) and formed only two new leaves. Plants were inoculated with active (AC) or heat-killed bacterial cells (HKC), to distinguish the role of active endophytic bacteria on plant nutrition. All biological treatments improved all parameters under evaluation, in comparison to MMN; for example, the number of new leaves was 6–7, and fresh weight biomass (root, and shoot fresh weight) was higher with *E. cloacae* AC (100 and 55% respectively). The statistical analysis indicates no significant differences between bacterial treatments. Interestingly the fresh weight was statistically different between active *E. cloacae* cells (2.09g ± 0.75g) and heat-killed *E. cloacae* (1.125g ± 0.25g); no differences were observed among the other bacterial treatments, but the fresh weight was lower for MMN treatment (−0.525g ± 0.63g). Another parameter that showed statistical difference was plant height when both active *E. cloacae* and *K. pneumoniae* were inoculated. Finally, no significant differences were observed among the treatments in respect to the number of leaves and root length. As expected, control plants that were only kept moist, without nutrients, died. After 60 days, only the plants inoculated with *E. cloacae* AC showed intracellular bacteria within root cells after 3,3 Diaminobenzidine (DAB) staining (Supplementary Figure S3). Plants inoculated with all other microbes were free of any evidence of internal colonization of bacteria.

### ^15^N Bacterial Transfer to Pheophytin

**FIGURE 6:**
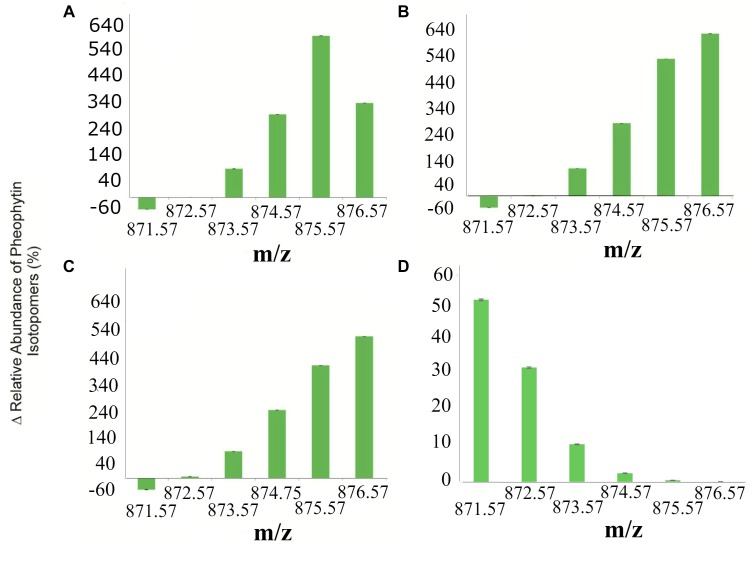
Isotopic enrichment of N atoms of banana plants pheophytin by ^15^N-transfer from bacteria. **(A)** Percentage increase of pheophytin isotopomers incorporation from plants inoculated with ^15N^*E. cloacae*. **(B)** ^15N^*K. pneumoniae*, **(C)** ^15N^*E. coli*, and **(D)** the average of all ^14N^bacterial treatments. The 871.57 *m/z* is the most abundant mass because there’s no ^15^N labeled into pheophytin. The data points are the mean values ± standard error of the mean from three independent experiments comparing ^14^N and ^15^N-labeled bacteria.

To determine the transference of ^15^N bacterial to banana plants, the content of ^15^N in pheophytin was measured. The analysis of the relative abundance of pheophytin isotopomers is shown in Figure 6. Those plants inoculated with ^14^N-labeled active cells (ACs) of *E. cloacae*, *K. pneumoniae*, and *E. coli* had a distribution of isotope peaks in agreement with the theoretical values of pheophytin formula (C_55_H_74_N_4_O_5_, [M+H]^+^ = 871.573). Then, incubation of banana plants with ^15^N-enrichment *E. cloacae*, *K. pneumoniae*, or *E. coli* resulted in incorporation of ^15^N into isotopomers of pheophytin.

The percentage of relative abundance of the isotopomers for *m/z* 872.576, 873.576, 874.575, 875.575, 876.575 was 0.07, 108.29, 313.78, 609.92 and 357.13% respectively (*p* < 0.2818; *p* < 0.2821; *p* < 0.0873; *p* < 0.0326; and *p* < 0.0224 respectively) ^14^N and ^15^N groups inoculated with *E. cloacae* AC (Figure 6A). The Figure 6B shows the percentage of relative abundance of ^15^N/^14^N in plants inoculated with active *K. pneumoniae* cells at *m/z* 872.576, 873.576, 874.575, 875.575, 876.575 was 2.37, 109.30, 287.66, 541.17, and 640.88% respectively (*p* < 0.0858; *p* < 0.0643; *p* < 0.1871; *p* < 0.0582; *p* < 0.0400; and *p* < 0.0213 for 871.576, 872.579, 873.581, 874.585, 875.590, and 876.594). For *E. coli*, the non-endophyte control, we found an increment of 6.40, 101.75, 258.05, 426.29, and 535.71% respectively (*p* < 0.4065; *p* < 0.6736; *p* < 0.2257; *p* < 0.0738; *p* < 0.0072; and *p* < 0.0208 respectively for *m/z* 871.575, 872.579, 873.582, 874.586, 875.589, and 876.631); data are showed in Figure 6C. Finally, the Figure 6D shows the average of the relative abundance of the isotopomers of MMN mineral solution and all bacterial treatments without labeling (containing ^14^N). Clearly, the abundance is dominant relative to base peak pheophytin with *m/z* 871.57. The ^15^N uptake to form isotopomer *m/z* 876.57 pheophytin was notably greater for all bacterial treatment in comparison with unlabeled bacteria, supporting the presence of ^15^N atoms into the tetrapyrrole ring of the pheophytin molecule. The abundance of the isotopomer 876.5 *m/z* was highest for inoculation with active *K. pneumoniae* and active *E. coli*, meaning ^15^N incorporation in the four nitrogen atoms of pheophytin. For *E. cloacae* AC, the isotopomer with more abundance was 875.575 m/z, indicating the incorporation of ^15^N in three of the four nitrogen atoms of pheophytin.

## Discussion

The association between plant host and microbial endophytes is known to influence individual plant fitness and plant population dynamics. In this study, we addressed the question of whether the native endophytes of the banana, which are antagonistic to the fungus *P. fijiensis*, may support the growth of host plants in nutrient poor soils by providing nitrogen to plants in two ways: (1) through the colonization of the root and later degradation by plant cells, or (2) through consumption of cellular remains directly from the soil. Two endophytic strains were selected, which were identified as *E. cloacae* and *K. pneumoniae*. Both bacteria were identified using proteomics and molecular methods. MALDI-TOF-MS analysis provided accurate resolution of bacterial identity at genus, species and subspecies levels.

We observed a 100% correlation between both techniques, however, as shown in Figure 2 the comparative analysis between the MALDI Biotyper databases and the mass spectrum protein profiles, shows at least four *Enterobacter* species (*asburiae*, *cloacae*, *hormaechei*, and *ludwiggi*) with a high score value between 2,190 and 2,290. Identification of species within the *E. cloacae* complex is often difficult using the 16s rRNA sequencing method. Here the combination of these methods allowed identification to *E. cloacae*. Based on the phylogenetic tree constructed with 16S rRNA, the strain clustered with other endophytic *E. cloacae* strains isolated from banana. For *RU1E* strain two species were proposed by the Biotyper database: *K. pneumoniae* and *K. variicola*. However, the highest score value (2,260) suggests the identification of *K. pneumoniae*. The molecular analysis supports the identification by mass spectrometry. Both species of bacteria have been isolated before as endophytes in banana plants cv. Cavendish Grand Naine’ and showed antagonistic activities toward *Fusarium oxysporum cubense* and capability for promotion of plant growth (Wang et al., 2010, 2012; Liu L. et al., 2016; Patel et al., 2018).

For a long time there has been concern about plant beneficial endophytes because they are closely related to human and animal opportunistic and pathogenic microbes (Rosenblueth et al., 2004; Mishra et al., 2017; Lima et al., 2018). Based on analyses of *in vitro* hemolysis of red blood cells and antibiotic resistance, we show that *K. pneumoniae* causes β-hemolysis and may be considered virulent, but *E. cloacae* has no hemolytic activity and is not likely virulent (Supplementary Figure S1). However, *E. cloacae* shows resistance to eight of twelve antibiotics (Supplementary Table S1), both methods are provided in Supplementary Material. In an ecological point of view, resistance to antibiotics of *E. cloacae* and *K. pneumoniae* might be an additional strategy to compete with other coexisting bacteria in soil or as endophyte.

### Morphological Characterization of Bacteria

Another feature studied here was the morphology of bacterial cells, not only as part of the identification protocol, but to reinforce our understanding of how these bacteria could interact physically with the host. Figure 3 highlights the formation of cell clusters in both *K. pneumoniae* and *E. cloacae*, and shows the presence of lateral flagella and pili after incubation log 18 h. In addition, Figure 3C showed *E. cloacae* cells surrounded by an extracellular material, like a mucus layer, and the formation of outer membrane vesicles (OMV’s). Nothing is known about the role of OMV’s for plant-endophyte cell–cell interactions. For mammalian and plant pathogens OMV’s modulate the immune system and are important for stress tolerance, virulence and tissue colonization (Ionescu et al., 2014; Bahar et al., 2016; Jan, 2017; Katsir and Bahar, 2017; Tan et al., 2018).

### Antifungal and PGP Traits of Microbes

Some endophytic bacteria have demonstrated the ability to inhibit pathogenic fungi from their host plants. It is believed that they compete for the niche, especially those that enter cells of plants. Here, both bacteria exhibit antagonistic activity against strains *Mf-1* and 102 of *P. fijiensis*. *K. pneumoniae* has a greater inhibitory activity against the two strains tested. Interestingly, *E. cloacae* has low antagonistic activity toward strain *Mf-1* which is a melanized strain, while strain 102 produces less melanin (Beltrán-García et al., 2014a). One possible explanation for the antagonistic effect may be the production of biosurfactants, in addition to other organic molecules and some hydrolytic enzymes, but more research will be necessary to unravel the mechanism of antagonism. Until now, it has only been reported that endophytes in genus *Bacillus* are antagonistic to *P. fijiensis* (Ceballos et al., 2012). A future scenario for agriculture is that many soils will have low nutrient content, high salinity, low moisture levels, and require expensive chemical fertilizers and other agrochemical inputs for crop production. Application of endophytic microbes may decrease dependence on chemical inputs and increase resilience of crops. Typical plant growth promotion (PGP) traits were evaluated in both bacteria using microbiological approaches (Table 1). According to these results, *E. cloacae* and *K. pneumoniae* are indicated to be diazotrophic or nitrogen-fixers. Both endophytes were capable of growth in N-free Norris medium, and Nfb medium showed the capacity to fix nitrogen. Growth in N-free medium suggests the ability of those endophytes to fix nitrogen from a microbiological approximation (Table 1). PCR amplification of the *NifH* gene as a biomarker and Acetylene Reduction Assay (ARA) are generally accepted as the most accurate test to determine diazotrophy, but some studies have shown high correlation between the molecular method, the chromatographic method and the growth on nitrogen-free media (Roesch et al., 2007; Radhapriya et al., 2018). Endophytes that can fix nitrogen and directly supply fixed nitrogen to the host will play a significant role in sustainable crop production. *Enterobacter* and *Klebsiella* have been commonly reported as endophytic diazotrophs in banana and other plants (Thomas et al., 2008; Thomas and Soly, 2009; Martínez-Rodríguez et al., 2014; Verma et al., 2018). Recently Liu et al. (2017) stated that *Paenibacillus*, *Enterobacter*, *Klebsiella* and *Pantoea* were not endophytic diazotrophs in the conventional sense, since they gained this trait through lateral transfer of genes. Siderophore production was observed in *E. cloacae* but not in *K. pneumoniae*. Siderophores are small chemical molecules that bacteria synthesize intracellularly, and can stimulate plant growth improving plant nutrition in terms of iron, zinc and other metals, and protection against phytopathogens. We observed a color change from blue to orange as indicative of the hydroxamate-type siderophore in the CAS tests suggests aerobactin production in this *E. cloacae* strain, but further studies are needed to identify the siderophore type. ACC deaminase is an enzyme responsible for cleavage of the plant ethylene precursor, impacting plant senescence. However, the ACC deaminase activity was negative for both strains, but not for the *E. coli* strain used as a non-endophyte.

Nitrogen loss and limited bioavailability of phosphorus are the main factors that restrict plant growth. It is known that endophytic bacteria solubilize inorganic phosphate by organic acid release. As shown in Table 1, *E. cloacae* and *K. pneumoniae* have phosphate solubilization indices of 148.59 ± 5.38 and 131.11 ± 7.84, respectively. Congruently, both genera have been previously reported to be phosphate solubilizers (Rajput et al., 2015; Dhole et al., 2016; Shabanamol et al., 2018; Zheng et al., 2018). The production of phytohormones such IAA is another factor influencing plant growth. Indole-3-acetic acid is the most abundant exogenous auxin, which has roles in stem elongation and root growth. Auxin-producer bacteria stimulate proliferation of lateral roots that increases nutrient absorbing surfaces and results in better assimilation of water and nutrients from the soil. *E. cloacae* was found to release an appreciable amount of IAA (74.2 μg/mL) after 120 h of incubation, which was induced by L-tryptophan (50 mM). Otherwise, the *K. pneumoniae* strain was a low auxin producer (0.328 μg/mL); this concentration is much lower compared to those reported in rhizospheric and free-living strains of *K. pneumoniae* (Sachdev et al., 2009; Rajput et al., 2015; Kuan et al., 2016).

### Plant Growth Support

In this study, we sought to understand whether endophytes might maintain the growth of banana plants in the absence of nutrients, and whether they influence plant development due to their PGP traits. Some studies have shown the potential of *E. cloacae* to be a plant growth promoter, but little is known about *K. pneumoniae* (Bhise et al., 2016; Liu W. et al., 2016). During *in vitro* inoculation with the two endophytes and the non-endophyte *E. coli* (as control), we observed that they support growth of banana plants compared to negative (water) and positive (MMN solution) controls. Plants treated with water or an MMN solution showed severe deterioration; water-treated plants died after 20 days, which is expected in soils without nutrients. Certainly, this treatment (just water) was carried out to determine if the native endophytes present in micropropagated plants might be able to exert effects on development and allow comparison to the other treatments. Plants in the MMN treatment survived the entire experiment, but loss of biomass was up to 30% from initial weight, only 2 slightly chlorotic leaves and the number of roots diminished 29%, which indicates that the plants did not have enough nutrients to produce or maintain roots and grow aboveground. Weekly, we applied active (AC) and heat-killed bacteria (HKC) to the plants. The HKC cells were incorporated into the soil to discriminate a purely nutritional factor with those of metabolically ACs and with colonization capacity. Increases in fresh weights and heights due to addition of AC and HKC bacteria was for *E. cloacae* 100 and 60%, for *K. pneumoniae* 55 and 38%, and for *E. coli* 55 and 50%, respectively. Nevertheless, there were no significant statistical differences for leaf emission and root lengths with biological treatments, but there were significant differences between each of the treatments and the MMN treatment (Figure 5). Growth parameters were not statistically significant between AC and HKC treatments for *E. coli* and *K. pneumoniae*. The little or no difference between microbial treatments suggests that bacteria can be consumed by plants in both states (active and inactive). If the active bacteria cannot colonize the roots internally, they will die in the soil, so the plant will later take the cellular remains and use them as a source of nutrients. As we show only *E. cloacae* was sustainably internalized into the root of the banana plant after 60 days post-inoculation. It was surprising to see that *E. coli* also stimulated plant growth. However, Nautiyal et al. (2010), in a study of *Zea mays*, used different strains of *E. coli* isolated from the soil, and found enhanced seedling growth and nutrient uptake. Here, the ATCC *E. coli* as we have shown was positive for nitrogen fixation, produced auxins, and surprisingly was positive for ACCd.

### ^15^N Tracking on Pheophytin Molecule

Decomposition of microbial proteins and other nutrients could explain biomass increases in plants treated with HKC and *E. coli* and is congruent with the concept that N-organic compounds such as proteins stimulate root formation and their morphology (Paungfoo-Lonhienne et al., 2008; Lonhienne et al., 2014a,b).

The incorporation of the ^15^N label into plants treated with ^15^N-labeled endophytes and *E. coli* is consistent with a scenario where N is transferred to plants from ^15^N-labeled bacteria when plants are limited in nutrients (Beltran-Garcia et al., 2014b). According to results shown in Figure 6, plants have ^15^N in pheophytin, a molecule derived from plant chlorophylls. The relative abundance of isotopomers indicates ^15^N label incorporation into some of the four nitrogen atoms of tetrapyrrole (1N = 873.57, 2N = 874.57, 3N = 875.57, and 4N = 876.57). The increment of 2N-4N signals is a definitive confirmation of N-transference from bacterium to plant tissues. Based on MS analysis, ^15^N transference from *K. pneumoniae* and *E. coli* was efficient since the four nitrogen atoms in pheophytin were labeled, while *E. cloacae* produced only three, even though this bacterium showed the best promotion of plant growth among these microorganisms. Additionally, we found that plants inoculated with *E. cloacae*, continued to show presence of *E. cloacae* within root cells after 60 days, while *E. coli* and *K. pneumoniae* could not be found within roots after that period, apparently, having been fully degraded (Supplementary Figure S2). One possible explanation for this discrepancy may be that in the rhizophagy symbiosis, *E. cloacae* may transport from the soil and transfer to plants primarily nutrients other than nitrogen, perhaps iron, zinc, magnesium, or other micronutrients, that are essential to growth and chlorophyll production, however, resistance to oxidative degradation, due to antioxidant production, enables the bacterium to protect its proteins from oxidative degradation (White et al., 2012, 2018). Paungfoo-Lonhienne et al. (2010), showed that *Arabidopsis thaliana* and tomato (*Lycopersicon esculentum*) take up the non-pathogenic bacterium *E. coli* and fungus *Saccharomyces cerevisiae* into root cells, they proposed the term ‘rhizophagy’ to explain that. Rhizophagy is considered a process in which live microbial cells are engulfed by root cells and partially digested to acquire the nutrients from microbes. We hypothesize that rhizophagy may account for at least some of the growth support we observed in our experiments. We further hypothesize that *E. cloacae* was the only microbe that was capable of establishing a long-term rhizophagy symbiosis with banana plants; and it may function in part by carrying nutrients acquired in soil into plants (White et al., 2018).

*Enterobacter cloacae* may be a keystone member of the microbial community of banana plants that serves as growth promoter and to confers tolerance to stress to plants (Yaish et al., 2015; Andrea-Barrao et al., 2017; Niu et al., 2017; Singh et al., 2017). Consistent with this, in other experiments we observed that endophytic strains of *E. cloacae* can modify genetic responses in the roots of banana and maize, mainly those genes that are involved in cell wall remodeling, response to oxidative stress, and nitrogen transporters (organic and inorganic), which allows the colonization and the uptake of nutrients from the of the soil (unpublished data).

## Conclusion

*Enterobacter cloacae* and *Klebsiella pneumoniae* isolates from roots and leaves of banana, potentially suppress the black Sigatoka fungus, and support plant growth in soils without organic matter. Our experiments suggest that all of the bacteria we tested in banana may be degraded and provide nutrients to plants, but only *E. cloacae* sets up a sustainable symbiosis in transference of nutrients to plants. Genetic studies will be realized to provide a better understanding of the mechanism involved in banana-*E. cloacae* interaction and could be further exploited to produce a biological agent to improve banana cultivation. *E. cloacae* and *K. pneumoniae* are classified as potential human pathogens. Further analyses will be necessary to determine whether the strain of *E. cloacae* we isolated from banana plants is pathogenic to humans before field experiments may be conducted.

## Author Contributions

GM-R performed the experiments, data processing and interpretation, and statistical analysis. BV-S contributed to data analysis, data interpretation, and writing the manuscript. FP and KP performed the experiments of SEM, data interpretation, and revising of the manuscript. LY and MK performed analysis of pheophytin isotopomers and data interpretation. BC-C contributed to data analysis, data interpretation of antagonistic activities, and revised the manuscript. MC-B revised the manuscript and interpreted the data. PDM contributed to data analysis, data interpretation and analysis tools, and revising of the manuscript. JW revised the manuscript with contributions and discussion of the rhizophagy process. MB-G conceived and designed the experiments, analyzed and interpreted the data, and wrote the manuscript.

## Conflict of Interest Statement

The authors declare that the research was conducted in the absence of any commercial or financial relationships that could be construed as a potential conflict of interest.
