# Supplementary material for: Enterobacter cloacae, an Endophyte That Establishes a Nutrient-Transfer Symbiosis With Banana Plants and Protects Against the Black Sigatoka Pathogen
Source: Front Microbiol. 2019 May 7;10:804. doi: 10.3389/fmicb.2019.00804 (PMC6513882; doi:10.3389/fmicb.2019.00804)
Supplement: Supplementary file 1 [file Data_Sheet_1.docx]

Supplementary Material

**Enterobacter cloacae, endophyte antagonistic to the black Sigatoka disease pathogen, establishes a nutrient-transfer symbiosis with banana plants under nutrient limitation.**

Gloria M. Macedo-Raygoza^1,2^, Benjamín Valdez-Salas^1^, Fernanda M. Prado^3^, Katia R. Prieto^3^, Lydia F. Yamaguchi^4^, Massuo J. Kato^4^, Blondy B. Canto-Canché^5^, Monica Carrillo-Beltrán^1^, Paolo Di Mascio^3^, James F. White^6^, Miguel J. Beltrán-García^2*^.

Correspondence: Miguel J. Beltrán-García, jbeltran@edu.uag.mx

**Hemolysis assay**


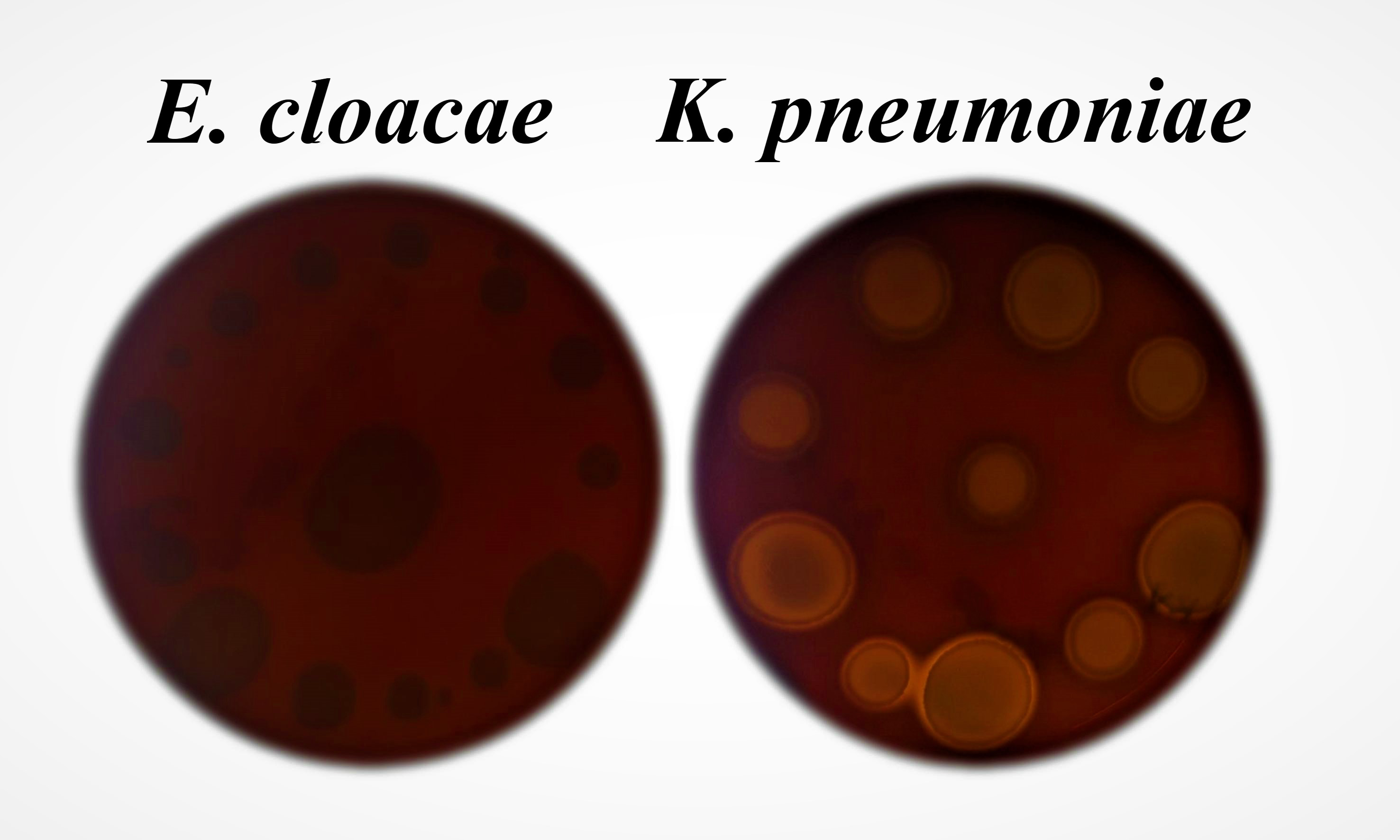
To detect if *E. cloacae* and *K. pneumoniae* are potentially virulent, their ability to produce hemolysis was evaluated. First, strains were inoculated in TSA medium and incubated for 18 h, at 30°C. Bacterial cells were suspended in 10mL of steril glucose solution (0.05%) at OD_600_= 0.2. Bacterial suspension was spotted in BD Columbia Agar with 5% Sheep blood and incubated for 24h at 30°C. Clear zones around the bacterial colonies indicate β-hemolysis, whereas green halos suggest α-hemolysis.

**Fig. S1.** Hemolytic activity test on *E. cloacae* and *K. pneumonia* strains isolated from banana tissues. Columbia Agar plates supplemented with 5% Sheep blood (BD) were inoculated with *E. cloacae* no produces hemolysis, meanwhile *K. pneumoniae* was positive for β-hemolysis, suggesting that *K. pneumoniae* is potentially pathogenic.

**Antibiotic Susceptibility Test**

To determine sensitivity or resistance to antimicrobial agents, fresh cultures of *E. cloacae* and *K. pneumoniae* were inoculated into TSA medium and incubated for 18 h at 30°C. Bacterial cells were suspended into 10 mL of sterile glucose solution (0.05%) at OD_600_ = 0.2. Then, Mueller-Hinton agar plates were swabbed with these bacterial suspensions and antibiotic multi disks (Antibiotic PT-35 Gram negative series 2, Investigación Diagnóstica, Mexico City, Mexico) were placed onto inoculated plates under a sterile environment. The plates were incubated at 30ºC for 24h. The antibiotics tested were: Amikacin (AK, 30μg), Ampicillin (AM, 10μg), Carbecillin (CB, 100μg) Cefotaxime (CFX,30μg), Ciprofloxacin (CPF,5μg), Chloramphenicol (CL,30μg), Gentamicin (GE, 10μg), Netilmicin (NET, 30μg), Nitrofurantoin (NF, 300μg), Norfloxacin (NOF, 10μg), and Sulphamethoxazole-Trimethroprim (SXT, 25μg). These antibiotics were chosen based on their use and availability to control Gram Negative infections in Mexico.

The criterion to determine resistance or susceptibility was based on the visual analysis of the formation of a halo for susceptibility and without or slightly halo formation for resistance, as shown in Figure S2 and Table S1.


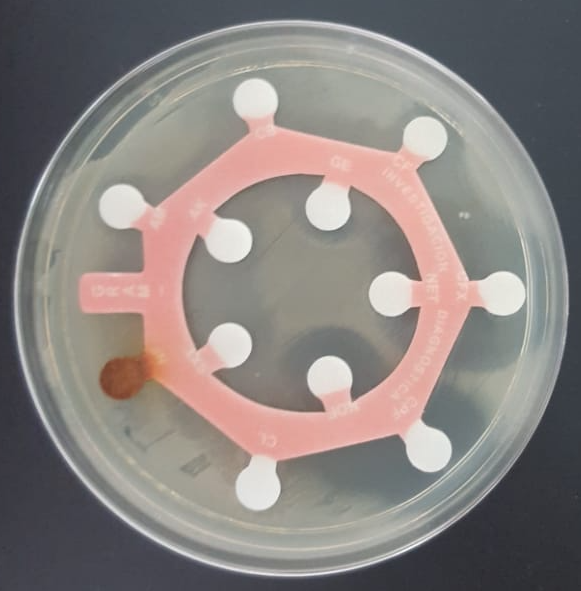


**Figure S2.** Antibiogram of *E. cloacae*, shows a marked inhibition zone in CFX (30 μg), CPF (5 μg), NOF (10 μg) and STX (25 μg). The inhibition zone for GE (10 μg) was slightly.

**Table S1.** Antimicrobial susceptibility test using agar disc diffusion for *K. pneumoniae and E. cloacae* Gram Negative strains

| **Antibiotic Gram Negative** | ***E. cloacae*** | ***K. pneumoniae*** |
| --- | --- | --- |
| Amikacin | **R** | **S** |
| Ampicillin | **R** | **S** |
| Carbencillin | **R** | **S** |
| Cefalotin | **R** | **S** |
| Cefotaxime | **S** | **S** |
| Ciprofloxacin | **S** | **S** |
| Chloramphenicol | **R** | **S** |
| Gentamicin | **R** | **S** |
| Netilmicin | **R** | **S** |
| Nitrofurantoin | **R** | **S** |
| Norfloxacin | **S** | **S** |
| Sulfamethoxazole/ Trimethoprim | **S** | **S** |

**DAB-Aniline blue staining to evidence root colonization**


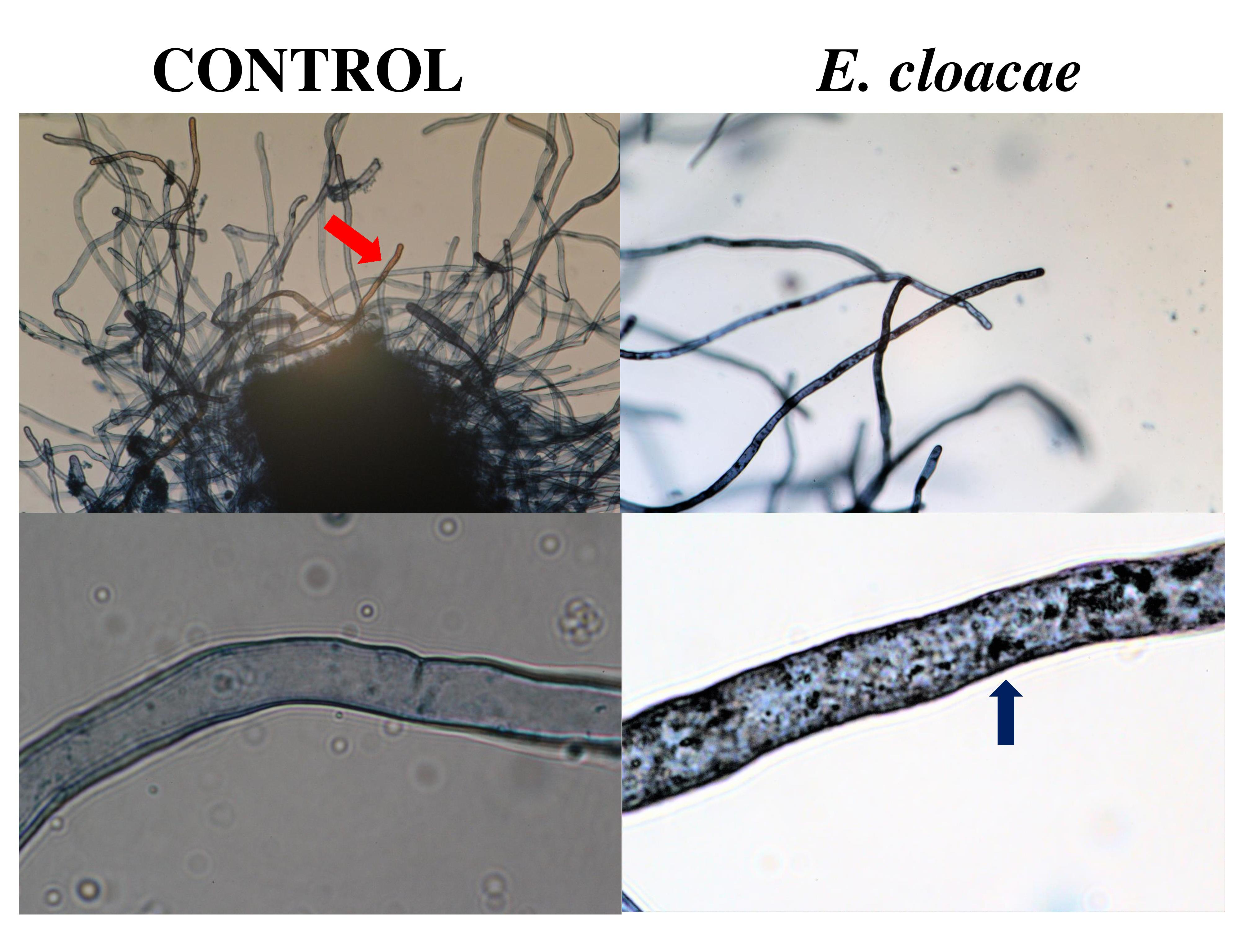
To visualize colonization into banana roots by *E. cloacae*, banana plants were inoculated with 10 mL of bacterial suspension adjusted to OD_600nm_ of 1. Roots were collected after 24, 48, 72, 144 h and 15 and 60 days post- inoculation. Plant roots were washed to eliminate sand and were put into 100 mL of 3mM DAB (3,3-diaminobenzidine tetrachloride) solution for 12-16 h. Afterwards the roots were excised and placed on an aniline blue solution for 15min. Roots were washed and put into a slide to be observed into the microscope (White et al., 2012).

**Figure S3. Colonization by *E. cloacae* of banana root hairs. For observations the tissues were stained with DAB-Aniline blue.** Plants with MMN treatment do not show intracellular bacteria into the root hairs (control), but there is presence of H_2_O_2_ in the hair roots (red arrow). The plant inoculated with *E. cloacae*, shows intact bacteria (blue arrow) as dark spot within the root hairs. Before 60 days the intracellular root colonization was negative.
